# Inhibition of lncRNA DILC attenuates neuropathic pain via the SOCS3/JAK2/STAT3 pathway

**DOI:** 10.1042/BSR20194486

**Published:** 2020-06-17

**Authors:** Yujie Liu, Lu Feng, Shichao Ren, Yingxiu Zhang, Jing Xue

**Affiliations:** 1 Department of Anesthesiology, The Second Affiliated Hospital of Tianjin University of Traditional Chinese Medicine, Tianjin 300150, China; 2 Department of Orthopedics, Qingyang People's Hospital, Qingyang 745000, Gansu Province, China; 3 Department of Anesthesiology, Qingyang People's Hospital, Qingyang 745000, Gansu Province, China

**Keywords:** inflammation, lncRNA DILC, neuropathic pain, SOCS3, the JAK2/STAT3 pathway

## Abstract

Long noncoding RNAs (lncRNAs) have been involved in the development of multiple pathological processes including neuropathic pain. The aim of the present study is to investigate the role of lncRNA down-regulated in liver cancer stem cells (DILC) in the progression of neuropathic pain and its underlying mechanism. Neuropathic pain rat model was established with the bilateral chronic constriction injury (bCCI) method. The results from quantitative PCR analysis in the spinal cord showed that DILC was significantly up-regulated in rats with bCCI compared with the sham group. DILC down-regulation mediated by intrathecal administration of DILC siRNA significantly increased the mechanical shrinkage threshold (MWT) and paw withdrawal threshold latency (PWTL), decreased the positive frequency for nerve sensitivity to cold and suppressed the expression of inflammatory genes in bCCI rats. Down-regulation of DILC induced suppressor of cytokine signaling (SOCS3) expression and inhibited the phosphorylation of signal transducer and activator of transcription 3 (p-STAT3) in spinal cord tissues. Western blotting showed that down-regulation of DILC by DILC siRNA transfection induced SOCS3 expression and inhibited the expression of p-Janus kinase 2 (p-JAK2) and p-STAT3 and their downstream genes in primary microglia*.* Furthermore, down-regulation of DILC increased the viability of primary microglia, suppressed apoptosis, and inhibited the production of interleukin (IL)-6 and IL-1β in microglia. In contrast, overexpression of DILC showed the opposite functions to those of DILC knockdown. In conclusion, silence of lncRNA DILC attenuates neuropathic pain via SOCS3-induced suppression of the JAK2/STAT3 pathway.

## Introduction

Neuropathic pain occurs from lesions or diseases affecting the somatosensory pathways within the peripheral or central nervous system (CNS). It is reported that neuropathic pain accounts for 7–8% prevalence among the general population [1]. Due to the complex mechanism, neuropathic pain is regarded as a major challenge to currently available clinical therapy of neurogenic pain syndromes. So, improving the understanding of the mechanisms underlying neuropathic pain might eventually lead to more specific, mechanism-oriented and thus more efficient treatment strategies [2].

Many studies have indicated that many intracellular signal pathways play important roles in the development of neuropathic pain. For example, a previous study showed that p38 played a critical role in microglial signaling under neuropathic pain conditions and represented a valuable therapeutic target for neuropathic pain management [3]. Inhibitors of the mitogen-activated protein kinase (MAPK) were regarded as potential antinociceptive drugs, even several substances have been advanced into clinical trials, such as Dimethyl Pimod [4]. Ma and Quirion reported that in several animal models of neuropathic pain, the extracellular regulated protein kinases (ERKs) were activated in neurons, microglia and astrocytes. The inhibitors of ERKs could alleviate pain at various time points [5]. Chemokine (C–C motif) ligand 2 (CCL2), a member of the CC chemokine family, was regarded as a trigger for spinal microglia activation. The CCL2 neutralizing antibody effectively prevented neuropathic pain behaviors following CCI [6]. Piotrowska et al. reported that modulation of CCL2/CCR2 pathway by microglial inhibitor as well as CCL2 receptor (CCR2) antagonist was effective for alleviating neuropathic pain development in rats [7]. Furthermore, the JAK2/STAT3 pathway was reported to be one of the most important cascades for the cellular transduction of signals in response to many pain modulators. Dominguez et al. reported that spinal nerve lesion led to an early activation of the JAK2/STAT3 pathway in the spinal cord microglia in projections areas of injured nerves. Blockade of this signaling pathway attenuated the generation of ipsilateral mechanical and thermal hypersensitivity and the mirror-image mechanical allodynia evoked by spinal nerve ligation [8].

Long noncoding RNAs (lncRNAs) are a number of non-coding RNAs that are >200 nucleotides and have been shown to participate in various cellular processes, such as cell proliferation, apoptosis, metastasis and inflammation [9–11]. LncRNAs are involved in many kinds of diseases, including cancers [12–14], cardiovascular diseases [15,16], immune diseases [17,18] and neurological diseases [19]. Many lncRNAs are highly expressed in the CNS, and their expression profiles are associated with specific neuroanatomical regions, cell types, or subcellular compartments, suggesting their potential functional roles in the nervous system [20]. A previous study showed that lncRNA down-regulated in liver cancer stem cells (lncRNA DILC) regulated behaviors of liver cancer stem cells via IL-6/STAT3 axis [21], so we supposed that lncRNA DILC might be associated with the progression of neuropathic pain through regulating STAT3 activation. The aim of study was to analyze the effect of lncRNA DILC on the progression of neuropathic pain and explore the underlying mechanism.

## Materials and methods

### Bilateral chronic constriction injury model of rats

Male Sprague-Dawley (SD) rats (250–300 g) were purchased from Better Biotechnology Co., Led (Nanjing, China) and kept in tray cages in the Animal Center of Tianjin University of Traditional Chinese Medicine (Tianjin, China). The rats were housed at 22°C under a 12/12 h light/dark cycle with free access to food and water. The CCI model was build according to the previous study [22]. Briefly, the rats were anesthetized by intraperitoneal injection of sodium pentobarbital at doses of 40–50 mg/kg. The sciatic nerve on each side was exposed through a mid-thigh incision and separation of the heads of the biceps femoris muscles. Then, each sciatic nerve was identified above the trifurcation and freed from surrounding loose connective tissue. Three snug ligatures of 4-0 chromic gut were placed (the space between the sciatic nerve ligatures is 1 mm) around the nerve of the experimental group (284 ± 22 g) and the sutures were placed with just enough pressure to produce mild blanching on the epineurium visible under the operating scope. Sham surgery was identical except that no ligatures were placed on the sciatic nerves of the control group (288 ± 24 g). A single individual performed all the sham and bilateral sciatic nerve ligations (*N* = 4). The protocol and procedure of the experiment were approved by the Institutional Animal Care and Use Committee of the Second Affiliated Hospital of Tianjin University of Traditional Chinese Medicine (Tianjin, China). All rats were killed by neck dislocation at 21 d after operation.

### Intrathecal administration

LncRNA DILC siRNA and scrambled control were obtained from GenePharma (Shanghai, China). For continuous administration, an intrathecal catheter was pre-implanted in each CCI model rat. Briefly, a 22 G needle (Beyotime Biotechnology, Shanghai, China) was inserted into the sheath of the lumbar spine. The tip of the needle was located in the L6-S1 (Lumbar vertebra 6-Sacrum 1) gap. The body of the needle and the spine of the rat were approximately 20°, through the muscle, ligamentum flavum and dura mater. The rats displayed tail flick, indicating that the needle had entered the sheath. The catheter was inserted through the gap on the needle body, with the direction parallel to the longitudinal axis of the spine. The catheter was inserted into approximately 4 cm, so that the tip of the catheter was located at the lumbar distention. Different concentrations of DILC siRNA and scrambled control were administered to CCI model rats using the pre-implanted intrathecal catheter. Briefly, 2 or 5 mg/kg DILC siRNA was administered intrathecally once daily for 4 days after CCI. As a control, 5 mg/kg scrambled siRNA was administered intrathecally at the same frequency.

### Pain threshold assessment

Pain threshold of sharp withdrawal threshold after mechanical stimuli (MWT) for rats was assessed using pain gauge measurement (von Frey, IITC, U.S.A.). Briefly, at days 0, 3, 7 and 14 following operation, the rats were acclimated in transparent plastic cages with wire mesh floor for 30 min. Plantar surface of each hind paw was applied pressure from below with the calibrated Electronic von Frey filament and held for approximately 5 s. Then force applied at the time of sharp withdrawal was recorded.

*Thermal hyperalgesia*: Heat sensitivity was measured using paw withdrawal threshold latency (PWTL) in response to radiant heat. Briefly, at days 0, 3, 7, and 14 following operation, a radiant heat source beneath a glass floor was aimed at the plantar surface of the hind paw. The latency for each hind paw in each test was repeated for three times. The hind paws were tested alternately with greater than 3 min intervals between consecutive tests. The three measurements of latency per side were averaged. The cutoff time was set at 20 s to avoid tissue damage.

*Cold allodynia*: At days 0, 3, 7 and 14 following operation, 0.1 ml acetone was gently sprayed onto the plantar surface of the hind paw using a multistepper pipette (Sigma) connected with a blunt rubber tube. Reactions performed with rapid withdrawal, licking, shaking or lifting of the hind paw after the spread of the acetone over the planter surface were regarded as positive. Measurement was performed three times for each hind paw with an interval of approximately 2 min between consecutive tests.

### Transfection

Primary microglia were isolated and cultured according to previous study described [23]. The cells were cultured in DMEM medium containing 10% fetal bovine serum (FBS). The pcDNA-DILC and pcDNA3.1 empty vectors were constructed purchased and from GenePharma Company. The pcDNA-DILC (1 µg/ml) and DILC siRNA (40 nM) and their relative controls were transfected into cells according to the instruction of Lipofectamine 3000 (Thermo) for 48 h.

### Real-time PCR

Total RNA was extracted from spinal cord tissues of rats or from microglia by using the Trizol Reagent (Thermo Scientific, Rockford, U.S.A.). The complementary DNA (cDNA) was synthesized with PrimeScript RT-PCR Kit (TaKaRa, Dalian, China). The real time PCR was performed according to the instruction of SYBR Premix Ex Taq Kit (TaKaRa). The reaction was run in ABI7500 Real-time PCR system (Applied Biosystems, Carlsbad, CA). The reaction program was as follows: 95°C for 5 min for initial denaturation, and followed by 40 cycles at 95°C for 10 s and 60°C for 34 s. All primers used in the present study were synthesized from Sangon Biotech (Shanghai, China). The relative expression of gene was normalized to the 18S RNA and quantified with the 2^−△△CT^ method.

### Western blot

The total protein was extracted by using protein lysis buffer from the spinal cord tissues. The protein concentration was measured using BCA Protein Kit (Thermo Fisher). The protein sample (20 µg) was separated on 10% sodium dodecyl sulfate/polyacrylamide gel electrophoresis (SDS/PAGE) and then transferred to polyvinylidene fluoride (PVDF) membranes. The blots were probed with rabbit anti-SOCS3 (Abcam, Cambridge, U.K.; 1:200 dilution), rabbit anti-pSTAT3 (Abcam, 1:1000 dilution), rabbit anti-STAT3 (Abcam, 1:1000 dilution), rabbit anti-pJAK2 (Abcam, 1: 1000 dilution), rabbit anti-JAK2 (Abcam, 1:5000 dilution) for overnight at 4°C. The blots were incubated with horse radish peroxide-conjugated secondary antibody. The imaging was performed with electron chemiluminescence (ECL) emitting solution. Finally, the blots were visualized with an Immobilon Western Chemiluminescent HRP Substrate system (Millipore Corp., Billerica, MA, U.S.A.).

### Cell Counting Kit-8 cell viability assay

Cell viability was measured by Cell Counting Kit-8 (CCK-8) assay (Dojindo, Kumamoto, Japan). After cells transfected for 48 h, 10 µl of CCK-8 was added to each well and incubated at 37°C for 1 h. Cell viability was measured at 450 nm using a microplate reader.

### Flow cytometry

After transfected with pcDNA-DILC or DILC siRNA for 48 h, cells were harvested and re-suspended in 500 µl of binding buffer and then mixed with 10 µl of Annexin V (Beyotime Institute of Biotechnology, China) for 15 min in the dark at room temperature. Cells were added with 5 µl of propidium iodide (PI; Beyotime Institute of Biotechnology) and incubated for 15 min. Finally, samples were analyzed using a FACSAria flow cytometry (BD Biosciences, San Jose, CA, U.S.A.).

### Enzyme-linked immunosorbent assay

The production of interleukin-6 (IL-6), IL-1β, and tumor necrosis factor-α (TNF-α) in spinal cord tissues and cell culture supernatants were measured according to instructions of their relative enzyme-linked immunosorbent assay (ELISA) kits.

### Statistical analysis

The data were analyzed by using SPSS 22.0 software. All the measurements from at least triplicates are presented as mean ± SD. The statistical analysis was performed with one-way analysis of variance, followed by Dunnett's test for multiple comparison to a control group. *P* < 0.05 was considered statistically significant.

## Results

### LncRNA DILC was significantly up-regulated in rats with bCCI

**Figure 1:**
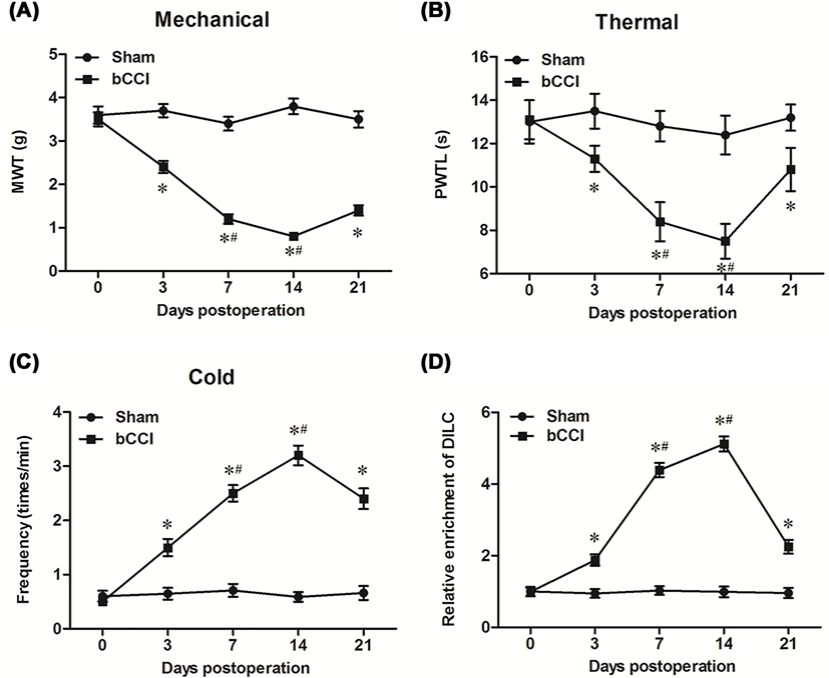
DILC was significantly up-regulated in rats with bCCI The bCCI model was built in SD rats. Pain threshold of rats to mechanical, thermal and cold stimulation were measured before bCCI surgery and then at different postoperative time points. (**A**) Mechanical allodynia; (**B**) thermal hyperalgesia; (**C**) cold hyperalgesia. (**D**) The expression of DILC was measured by real-time qPCR. *N* = 4, *P<0.05 *vs.* sham group.

First, we checked the expression profile of lncRNA DILC in the CNS at different developmental stages. The results showed that DILC level was relatively low in the CNS of rat embryo, and moderate in CNS of new born individuals; in the CNS of rat after born (both new born and adult), DILC displayed higher expression level in the spine than the brain; it was quite interesting that, the level of spinal DILC was much higher than cerebral DILC in adult rats, but the difference in DILC levels between the brain and spinal cord is not so outstanding in newborn rats (Supplementary Figure S1). Moreover, we investigated its expression in main cell types in adult rats, and the results showed that was mainly expressed in microglia (Supplementary Figure S2). To analyze whether DILC was involved the progression of neuropathic pain, we investigated the expression levels of DILC in spinal cord tissues. The results of neuropathic pain threshold assay showed that MWT and PMTL were significantly decreased in bilateral chronic constriction injury (bCCI) rats compared with sham group (*P*<0.05, Figure 1A,B). Besides, the positive frequency for nerve sensitivity to cold was dramatically increased in bCCI rats compared with sham group (*P*<0.05, Figure 1C). These results suggested that the rat model of bCCI was successfully built. The result of real-time qPCR showed that DILC was significantly increased in bCCI rats after 3, 7, 14 and 21 days, and it reached the peak at day 14 post-operation (*P*<0.05, Figure 1D).

### DILC siRNA alleviated the neuropathic pain of bCCI rats *in vivo*

**Figure 2:**
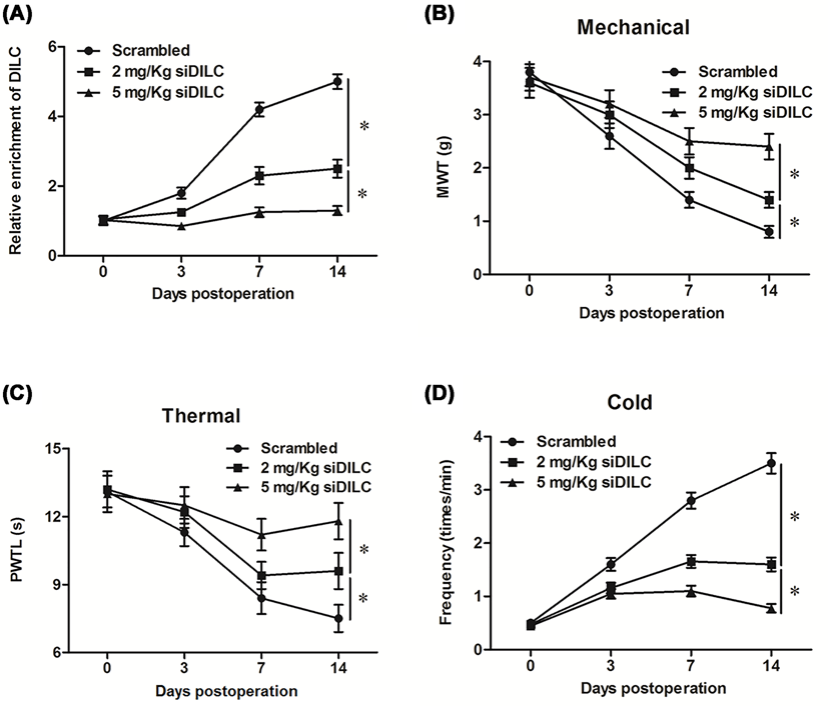
DILC siRNA alleviated the neuropathic pain of bCCI rats Different concentrations of DILC siRNA and the scrambled control were administered to rats using the pre-implanted intrathecal catheter. (**A**) The expression of DILC in each group was measured by real-time qPCR. Pain threshold of rats to mechanical, thermal and cold stimulation were then measured: (**B**) thermal hyperalgesia; (**C**) thermal hyperalgesia; (**D**) cold hyperalgesia. *N* = 4, *P<0.05 *vs.* scrambled group.

To further analyze the effect of DILC siRNA on the neuropathic pain of bCCI rats *in vivo*, lncRNA DILC siRNA or scrambled siRNA (scramble) was administered to rats by using the pre-implanted intrathecal catheter. The results showed that the expression level of DILC was significantly decreased in bCCI rats administrated with 2 or 5 mg/kg of DILC siRNA (siDILC) (*P*<0.05, Figure 2A). Furthermore, MWT and PMTL were significantly increased in bCCI rats with 2 or 5 mg/kg of siDILC intrathecal administration, and the threshold up to maximum at day 14 post operation compared with the scramble group (*P*<0.05, Figure 2B,C). The positive frequency for nerve sensitivity to cold was dramatically decreased in bCCI rats with 2 or 5 mg/kg of siDILC intrathecal administration, also decreased to minimum at day 14 (*P*<0.05, Figure 2D). These results indicated that DILC had an important role in the progression of neuropathic pain.

### DILC siRNA inhibited inflammation in rats with bCCI *in vivo*

**Figure 3:**
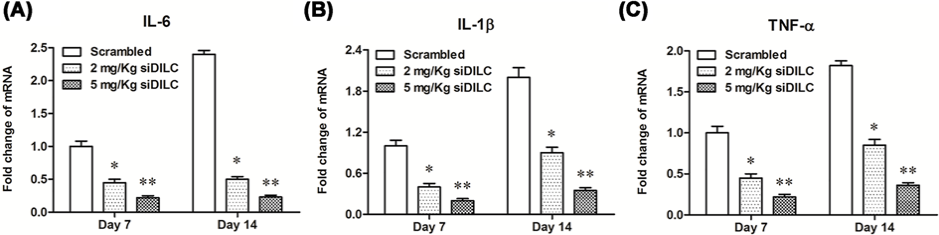
DILC siRNA inhibited inflammation in rats with bCCI After different concentrations of DILC siRNA and scrambled control were administered to rats for different time, the production of (**A**) IL-6, (**B**) IL-1β and (**C**) TNF-α in spinal cord tissues was measured according to instructions of their relative ELISA kits. *N* = 4, *P<0.05 *vs*. scrambled group.

To further investigate the effect of DILC on the inflammation of bCCI rats, the expression level of IL-6, IL-1β and TNF-α in spinal cord tissues was measured by real-time qPCR. The results showed that the expression of IL-6, IL-1β and TNF-α was dramatically decreased when bCCI rats were intrathecal administrated with 2 mg/kg siDILC or 5 mg/kg siDILC, and they all decreased to the minimum at day 14 (*P*<0.05, Figure 3A–C). These results suggested that DILC siRNA inhibited inflammation in rats with bCCI.

### DILC siRNA induced SOCS3 expression and inhibited the phosphorylation of STAT3

**Figure 4:**
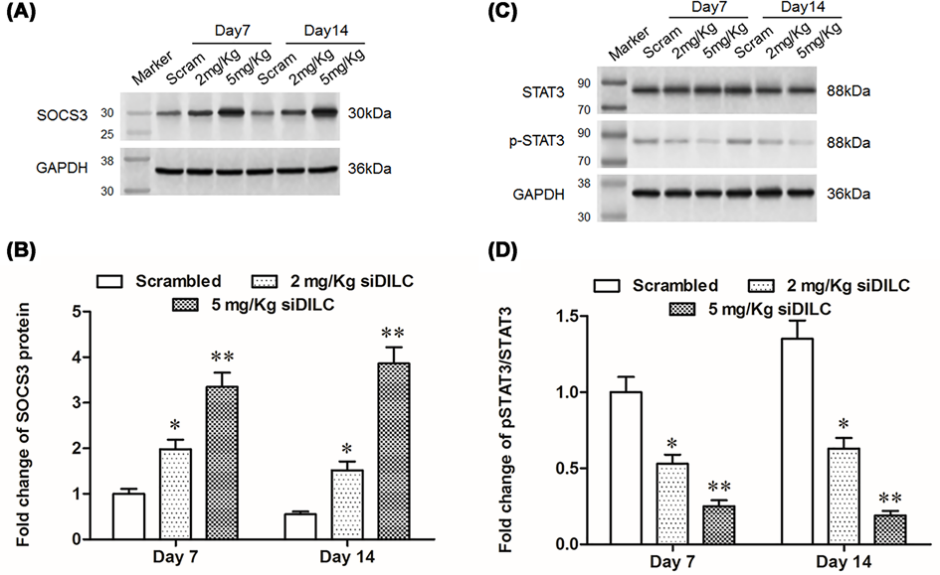
DILC siRNA induced SOCS3 expression and inhibited the phosphorylation of STAT3 After different concentrations of DILC siRNA and scrambled control were administered to rats for different time, the expression of SOCS3, pSTAT3, STAT3 was measured by Western blotting. (**A,B**) The expression level of SOCS3; (**C,D**) the expression of pSTAT3 and STAT3. *N* = 4, *P<0.05, **P<0.01 *vs.* scrambled group.

The further study showed that the expression of SOCS3 was significantly increased when bCCI rats were intrathecal administrated with 2 mg/kg siDILC or 5 mg/kg siDILC at day 7 and day 14 compared with scrambled group (*P*<0.05, Figure 4A,B). As the downstream gene of SOCS3, the level of the phosphorylation of signal transducer and activator of transcription 3 (p-STAT3) was noticeably decreased in bCCI rats intrathecally administrated with 2 mg/kg siDILC or 5 mg/kg siDILC (*P*<0.05, Figure 4C,D).

### DILC siRNA inhibited the SOCS3/JAK2/STAT3 pathway *in vitro*

**Figure 5:**
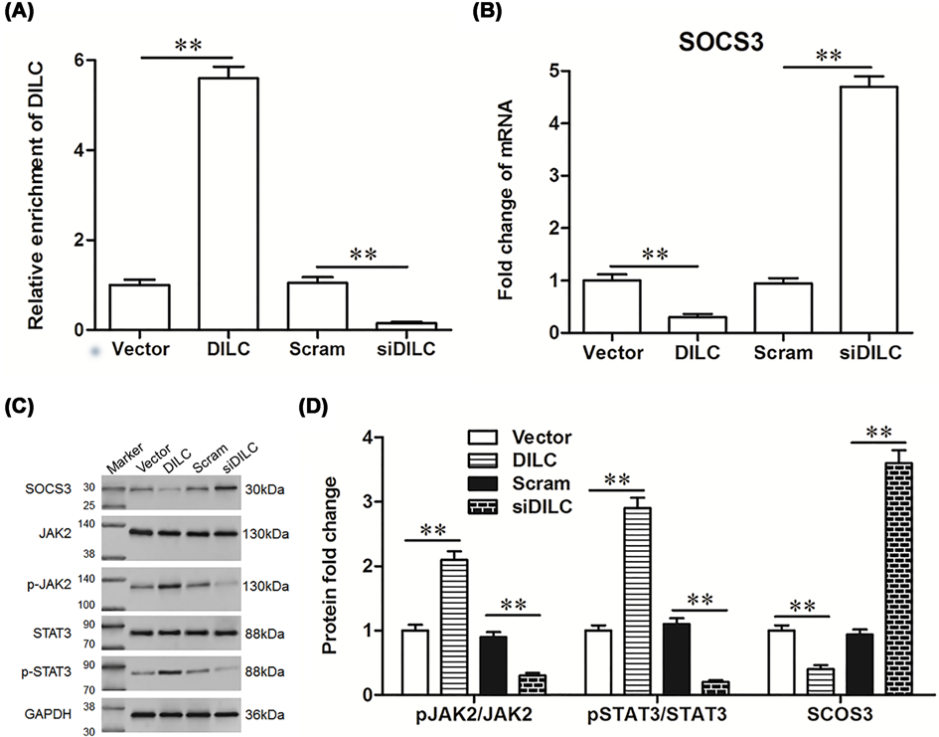
DILC siRNA inhibited the SOCS3/JAK2/STAT3 pathway *in vitro* The primary neurons and microglia were isolated and co-cultured and were transfected with pcDNA-DILC, DILC siRNA and their relative controls. (**A**) The expression of DILC was measured by real-time qPCR. (**B**) The expression of SOCS3 was measured by real-time PCR. (**C,D**) The expression of SOCS3, p-JAK2, JAK2, p-STAT3, STAT3 was measured by western blot. *N* = 4, **P<0.01 *vs.* vector group or scram group.

**Figure 6:**
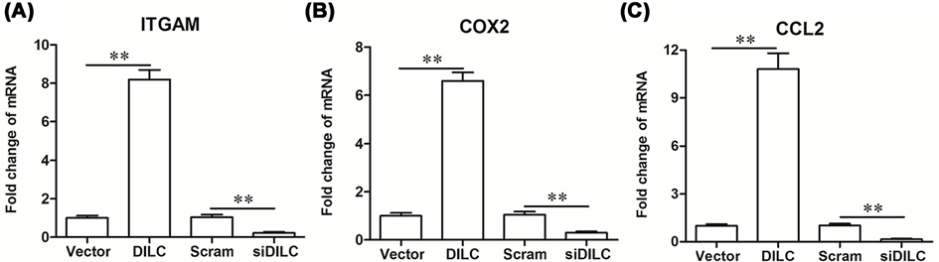
DILC siRNA inhibited the expression of down-stream genes of STAT3 After the primary neurons and microglia cells were transfected with pcDNA-DILC, DILC siRNA and their relative controls for 48 h, the expression of downstream of genes of STAT3 was measured by Real-time qPCR including (**A**) ITGAM; (**B**) COX2; (**C**) CCL2. *N* = 4, **P<0.01 *vs.* vector group or scram group.

To further analyze the effect of DILC on the SOCS3/JAK2/STAT3 pathway *in vitro*, primary microglia cells were isolated and cultured, and pcDNA-DILC, DILC siRNA and their relative controls were transfected into the cells. The results showed that the expression of DILC was significantly increased after transfection with pcDNA-DILC, and dramatically decreased after transfection with DILC siRNA (*P*<0.05, Figure 5A). The expression of SOCS3 was significantly down-regulated by pcDNA-DILC transfection, and was up-regulated by DILC siRNA transfection compared with relative controls (*P*<0.05, Figure 5B,C). Furthermore, the expression of pJAK2 and pSTAT3 was considerably up-regulated by pcDNA-DILC transfection, whereas they were down-regulated by DILC siRNA transfection (*P*<0.05, Figure 5C,D). These results indicated that down-regulation of DILC induced SOCS3 expression and suppressed JAK2/STAT3 signaling pathway *in vitro.* We further analyzed the effect of DILC on the expression of down-stream gene of STAT3 *in vitro*, the results showed that pcDNA-DILC significantly increased the expression of the downstream genes of STAT3, including integrin alpha M (ITGAM), cyclooxygenase 2 (COX2) and CCL2, whereas DILC siRNA dramatically reduced the expression of ITGAM, COX2 and CCL2 compared with relative controls (*P*<0.05, Figure 6A–C).

### LncRNA DILC siRNA increased the viability of microglia cells, and suppressed cell apoptosis and inflammation

**Figure 7:**
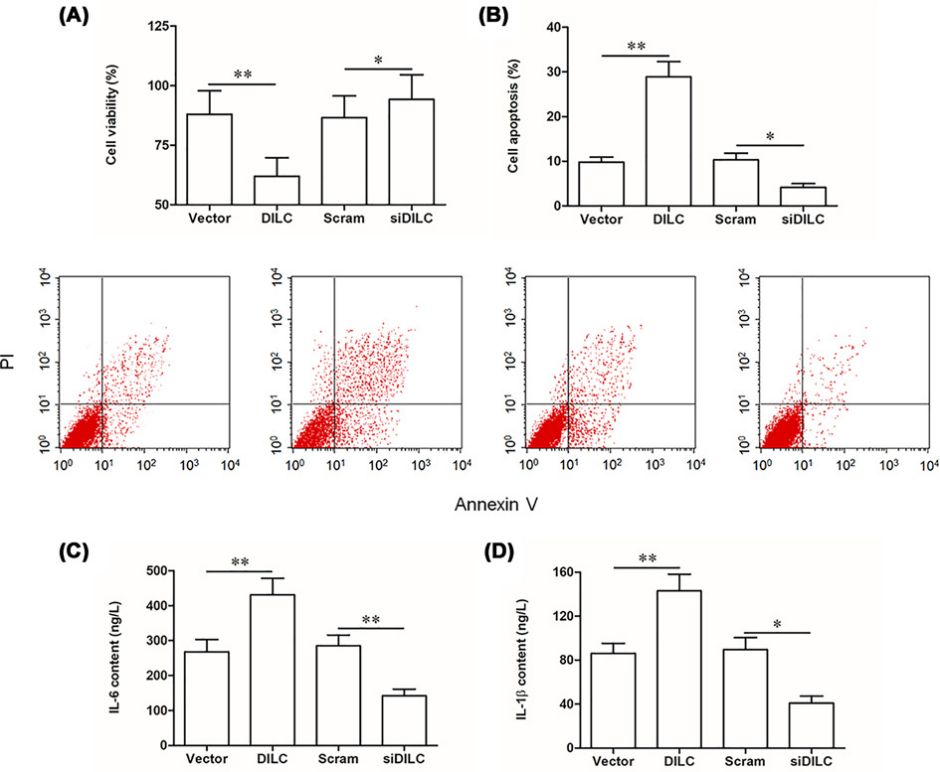
DILC siRNA increased the viability of primary microglia cells, suppressed cell apoptosis and inflammation Primary microglia were transfected with pcDNA-DILC, DILC siRNA and their relative controls for 48 h. (**A**) Cell viability was measured by CCK-8 assay. (**B**) Cell apoptosis was measured by flow cytometry. (**C**) The production of IL-6 was measured by ELISA. (**D**) The production of IL-1β was measured by ELISA. *N* = 4, *P<0.05 *vs*. vector group or scram group. **P<0.01 *vs.* vector group or scram group.

Then, the role of lncRNA DILC in regulating functions of microglia was investigated. Our results showed that cell viability was significantly decreased in pcDNA-DILC transfection group, whereas it was increased in DILC siRNA transfection group (*P*<0.05, Figure 7A). Flow cytometry assay showed that cell apoptosis was dramatically increased in pcDNA-DILC transfection group, whereas it was decreased in DILC siRNA transfection group (*P*<0.05, Figure 7B). Furthermore, pcDNA-DILC transfection significantly induced the production of IL-6 and IL-1β, whereas DILC siRNA reduced the production of IL-6 and IL-1β (*P*<0.05, Figure 7C,D). These results indicated that DILC siRNA increased the viability of microglia, and suppressed cell apoptosis and inflammation *in vitro*.

## Discussion

LncRNAs consist of a large fraction of the mammalian transcriptome. Increasing studies have revealed that the lncRNAs played important roles in the progression of various diseases, such as cancers [24–26], neurodegenerative diseases [27], respiratory diseases [28], cardiovascular diseases [16,29]. The present study demonstrated that lncRNA DILC was significantly increased in bCCI rats. DILC siRNA alleviated the neuropathic pain of bCCI rats and inhibited inflammation *in vivo*. Furthermore, DILC siRNA also inhibited the SOCS3/JAK2/STAT3 signaling pathway *in vivo* and *in vitro*.

SOCS3, a member of SOCS family, acted as feedback inhibitor of the JAK/STAT3 pathway, avoiding STAT3 phosphorylation. A previous study reported that SOCS3 expression in neurons caused a negative regulatory effect on signal transduction and STAT3 activation, which consequently contributed to excitotoxic neuronal death *in vitro*. SOCS3 could binds to gp130, a common signal transducing subunit with IL-6, or to JAK1 and JAK2, to inhibit signal transduction thus leading to a negative regulation of neuronal survival and axon regeneration *in vivo* and *in vitro* [30]. Furthermore, the antagonists or blocking drugs of JAK2/STAT3 signal pathway was reported to be concerned with the formation of neuropathic pain, such as aspirin-triggered Lipoxin A4 [31], procaine [32], WP1066 [33]. The present study showed that DILC siRNA induced SOCS3 expression and inhibited the JAK2/STAT3 pathway. It suggests that DILC siRNA alleviated the neuropathic pain of bCCI rats through inducing SOCS3 expression and inhibiting the JAK2/STAT3 pathway.

Microglia represent for 5–10% of glia in the CNS and are often considered resident macrophages. Microglia are activated after nerve injury which produce various chemical mediators, including proinflammatory cytokines that can produce immunological actions and can also act on neurons to alter their function [34]. The appropriate activation of microglia is beneficial for the body, and their apoptosis was reported to be appeared in the progression of nerve injury [35,36]. The present study demonstrated that DILC siRNA increased the viability of microglia, suppressed cell apoptosis and inflammation. These results may indicate that DILC knockdown plays a protective role in the progression of neuropathic pain.

Interestingly, previous studies on the function of lncRNA DILC in development of diseases revealed that it was down-regulated during tumorigenesis and sepsis and functioned as a suppressor gene in cancer development and sepsis, which depends on its suppressive effect on STAT3 activation [21,37–39]. However, in our current study, we found that lncRNA DILC was up-regulated and STAT3 was activated after CCI and lncRNA DILC positively regulated the activation of STAT3 and STAT3-induced production of inflammatory cytokines. As a macrophage like cell type, microglia display different functions with most of the constituent histocytes but have many characteristics similar to immune cells. There have been a few of cases that a gene functioned diversely in microglia compared with in constituent histocytes. A typical example is that transforming growth factor β1, a master gene in regulating cell proliferation, epithelial–mesenchymal transition and fibrosis in most cell types, plays a negative role in immune cell proliferation and differentiation [40]. It was also reported that lncRNAs functioned diversely in microglia compared with in constituent histocytes. For example, the noncoding RNA activated by DNA damage (lncRNA NORAD, also known as LINC00657) functioned as a tumor suppressor gene in lung cancer [41], but it was up-regulated in CCI rats and contributed to neuroinflammation through inducing expression of COX2, TNF-α and IL-1β [42], which is quite similar to lncRNA DILC.

In conclusion, the present study reported that DILC siRNA alleviated the neuropathic pain through inducing SOCS3 expression and inhibiting the JAK2/STAT3 pathway. LncRNA DILC may be a potential target for the treatment of neuropathic pain.

## Supplementary Material

Supplementary Figures S1-S2

## Abbreviations

bCCI: bilateral chronic constriction injury
CCK-8: Cell Counting Kit-8
CNS: central nervous system
COX2: cyclooxygenase 2
ELISA: enzyme-linked immunosorbent assay
ERK: extracellular regulated protein kinase
ITGAM: including integrin alpha M
IL: interleukin
lncRNA: long noncoding RNA
p-STAT3: phosphorylation of signal transducer and activator of transcription 3
PWTL: paw withdrawal threshold latency
