# Supplementary material for: Inhibition of lncRNA DILC attenuates neuropathic pain via the SOCS3/JAK2/STAT3 pathway
Source: Biosci Rep. 2020 Jun 17;40(6):BSR20194486. doi: 10.1042/BSR20194486 (PMC7300282; doi:10.1042/BSR20194486)
Supplement: Supplementary Figures S1-S2 [file BSR-2019-4486_supp.pdf]

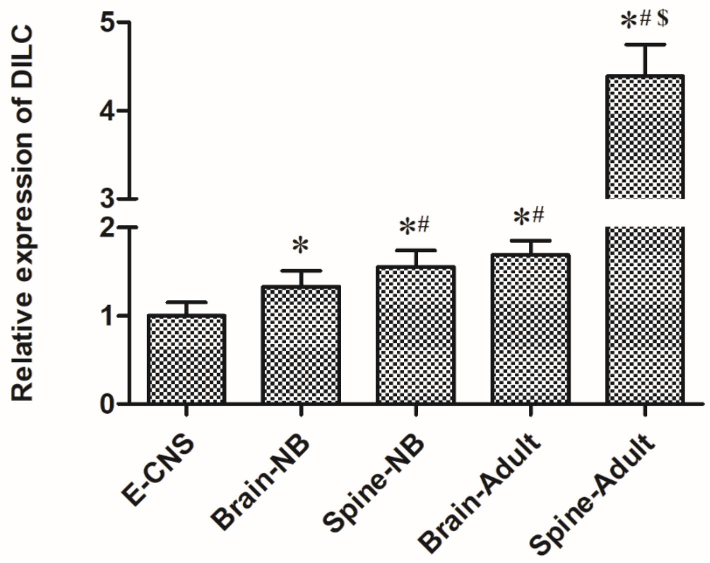

Relative expression of DILC
5
4
3
2
1
0
*# $
*
*#
*#
E-CNS
Brain-NB
Spine-NB
Brain-Adult
Spine-Adult

Supplementary figure 1. The expression pattern of lncRNA DILC in the rat CNS at different development stages. CNS: central nervous system;E: embryonic; NB: new born. E-CNS was sampled from the rat embryo at day14 of gestation.Brain-NB and Spine-NB weresampled from neonatal rats less than 3 days old. Brain-NB and Spine-NB weresampled from 6-month-old rats. *P< 0.05 vs. E-CNS; $^{\#}P$< 0.05 vs. Brain-NB; $^{\$}P$< 0.05 vs. Spine-NB.

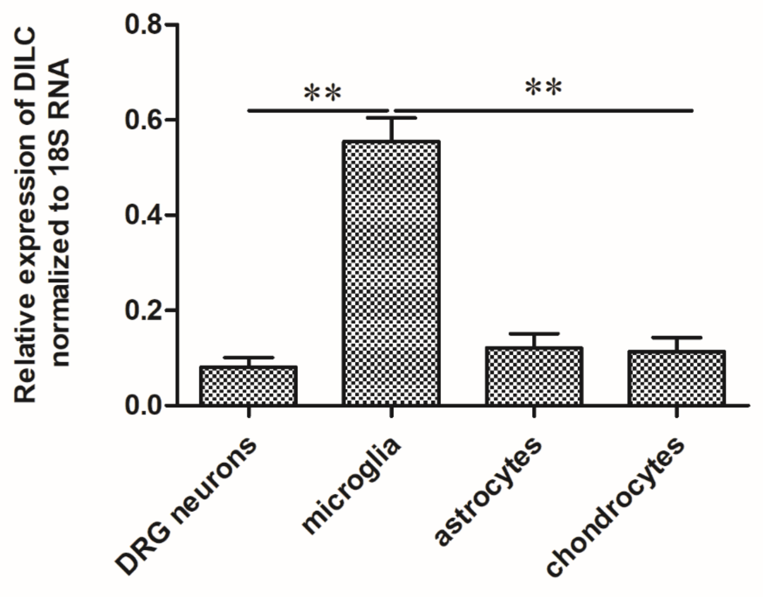
Relative expression of DILC normalized to 18S RNA
0.8
0.6
0.4
0.2
0.0
**
**
DRG neurons
microglia
astrocytes
chondrocytes

Supplementary figure 2. The expression pattern of lncRNA DILC in main cell types inadult rat spinal cord, including dorsal root ganglion (DRG) neurons, microglia, astrocytes and chondrocytes. **$P < 0.01$.
